# Replacing Soybean Meal with Sesame Meal in the Diets of Lactating Awassi Ewes Suckling Single Lambs: Nutrient Digestibility, Milk Production, and Lamb Growth

**DOI:** 10.3390/ani9040157

**Published:** 2019-04-11

**Authors:** Belal S. Obeidat, Rami T. Kridli, Kamel Z. Mahmoud, Mohammed D. Obeidat, Serhan G. Haddad, Hadil S. Subih, Mysaa Ata, Ahmed E. Al-Jamal, Tasneem Abu Ghazal, Jáfar Mansur Al-Khazáleh

**Affiliations:** 1 Department of Animal Production, Faculty of Agriculture, Jordan University of Science and Technology, Irbid 22110, Jordan; rkridli@just.edu.jo (R.T.K.); kmahmoud@just.edu.jo (K.Z.M.); mdobeidat@just.edu.jo (M.D.O.); shaddad@just.edu.jo (S.G.H.); aljamalahmed@yahoo.com (A.E.A.-J.); 2 Department of Nutrition and Food Technology, Faculty of Agriculture, Jordan University of Science and Technology, Irbid 22110, Jordan; hssubih@just.edu.jo (H.S.S.); tasnim.s92@hotmail.com (T.A.G.); 3 Department of Animal Production and Protection, Faculty of Agriculture, Jerash University, Jerash 26150, Jordan; mysaata@gmail.com; 4 Faculty of Agricultural Technology, Al–Balqa Applied University, P.O. Box 19117 Al–Salt, Jordan; jkhaza@bau.edu.jo

**Keywords:** Awassi ewes, sesame meal, nursing performance, intake and digestibility

## Abstract

**Simple Summary:**

Alternative feeds are agricultural or industrial by-products (rich in nutrient content) that can be used to replace part of the conventional ingredients in animal rations. These by-products are typically much lower in cost than conventional feeds and reduce the dependence on conventional feeds, which would increase the benefits and profitability of raising livestock. Another objective of this kind of experiments is to utilize agro-industrial by-products, such as sesame meal, which, in turn, would reduce their waste disposal and the associated pollution issues. Sesame meal can replace soybean meal at levels of 0%, 7.5%, and 15%. Milk production increased in SM-containing diets more than the control. Lamb growth improved in sesame meal-containing diets.

**Abstract:**

Two experiments were conducted to assess the influence of sesame meal (SM) feeding on nutrient digestibility, N balance, milk production and composition, ewes’ body weight change, and growth performance of lambs. In experiment 1, 18 ewe lambs were randomly distributed into three diets to evaluate the effects of soybean meal replacement with SM on nutrient intake, digestibility, and N balance. Treatments were no SM (SM0), 7.5% SM (SM7.5), or 15% SM (SM15) of the dietary dry matter (DM). Aside from intake and digestibility of ether extract (EE), which was greater in the SM-containing diets compared with SM0, intake and digestibility of the remaining nutrients was similar among dietary treatments. In experiment 2, 30 ewes suckling single lambs were randomly assigned to the same diets used in experiment 1. Intakes of DM, crude protein, neutral detergent fiber, and acid detergent fiber were unaffected by treatment. Milk yield was greater in SM diets than in the SM0 diet. Cost/kg of milk production decreased while feed efficiency improved in the SM-diets compared to the SM0 diet. In conclusion, results of the current studies demonstrate the possibility of replacing soybean meal with sesame meal in diets of lactating Awassi ewes.

## 1. Introduction

Soybean meal (SBM) is the most common source of protein in livestock diets in Jordan and worldwide. However, due to its high cost and fluctuation in price, livestock producers continuously search for alternatives with lower cost. Sesame meal (SM) is a nutritious by-product that shows good potential to replace the most expensive feed ingredient in livestock diets [1]. Sesame meal is one of the important residues of oil extraction from sesame seeds. The annual production of SM and sesame hulls in Jordan is 3600 tons [2] and 1 million tons globally [3]. Crude protein (CP) content in SM is high (46% CP), which makes it a good alternative for SBM [1]. Such substitution would reduce feed cost, and hence the cost of production. More research should address the use of SM in feeding sheep, especially during the early lactation period, where ewes are in a critical stage for the need of extra nutrients compared to other periods. Therefore, studies are needed to determine optimal levels of SM in the diet of nursing ewes. Several studies were conducted to evaluate the effect of feeding sesame meal or sesame hulls to lambs [1,4,5], kids [6,7], and dairy cows [8]. However, to our knowledge, no data are available on the effects of feeding SM to lactating ewes. The hypothesis of the current study is that using SM in diets of nursing ewes will yield similar performance to the use of SBM. If this hypothesis is proven, it will help in finding a protein source that can contribute to reducing the cost of rations, as well as reducing the cost of production. Therefore, two experiments were conducted to determine how SM would affect nutrient digestibility, N balance, lamb growth parameters, and milk production and composition when replacing SBM.

## 2. Materials and Methods

Two experiments were conducted at the animal research facilities of Jordan University of Science and Technology (JUST) after the approval of JUST Institutional Animal Care and Use Committee (#: 16/3/3/375). Before the commencement of the experiments, a veterinarian inspected the health of all experimental animals and udder status of the mature ewes. Animals continued to be routinely checked thereafter throughout the study. The two experiments were conducted to study the effect of replacing SBM with SM; nutrient digestibility and nitrogen balance were evaluated using ewe lambs in the first experiment (Exp. 1); milk production and composition and lamb growth were evaluated using ewes suckling single lambs in the second experiment (Exp. 2). Dietary treatments were (1) no SM (SM0), (2) 7.5% SM (SM7.5), or (3) 15% SM (SM15) of dietary dry matter (DM) in substitution of SBM. Sesame meal was purchased locally from one supplier. To limit animal selection and to ensure thorough mixing, sesame meal was sun dried and ground before being incorporated into the diets. The chemical composition of the SM and SBM was 91.1 and 90.5%, 46.3 and 46.2%, 15.8 and 14.3%, 8.5 and 9%, and 11.7 and 1.6% DM, CP, neutral detergent fiber (NDF), acid detergent fiber (ADF), and ether extract (EE), respectively. However, the metabolizable energy was 3.53 and 3.06 Mcal/kg for the SM and SBM, respectively. Diets were prepared immediately prior to the feeding period and were offered *ad libitum* with a 10% refusal level measured daily (Table 1).

### 2.1. Experiment 1

Eighteen Awassi ewe lambs (BW = 33.5 ± 0.69 kg; age = 6 to 7 months) were randomly assigned (6 ewe lambs/diet) into the three dietary treatments above. Lambs were ear tagged and weighed before the beginning of the experiment. The experiment lasted for 28 days; a 21-day adaptation period (animals housed in individual 0.75 × 1.5 m shaded pens) followed by a 7-day collection period (animals in 0.8 × 1.05 m metabolic cages). The shaded pens allowed *ad libitum* access to feed and water in plastic troughs.

Feeding, sampling procedure, and chemical analysis were carried out as described by Alshdaifat and Obeidat [9]. Briefly, feed refusals were weighed, recorded, and sampled to accurately measure nutrient intakes. At the end of the experiment refusals were analyzed for DM, CP, NDF, ADF, and EE. From days 22 to 28, ewe lambs were placed in metabolic cages equipped with fecal trays that allowed for total fecal and urinary collections. During this period, fecal output was collected and 10% was kept for chemical composition. At the same time, urine was collected using plastic containers and 5% was kept (−20 °C) to evaluate N balance. Each urine bottle contained 50 mL of 6 N HCL to prevent the losses of ammonia. At the end of the study, fecal and urinary samples were composited for each ewe lamb. Fecal samples were dried in a forced air oven at 55 °C to reach a constant weight and weighed to determine DM content. Fecal and dietary samples were then ground in a Wiley mill (Brabender OHG Duisdurg, Kulturstrase 51–55, type 880845, Nr 958084, Germany) to pass through a 1-mm screen and were stored in plastic bags at room temperature for subsequent analyses.

For N balance evaluation, the collected urinary samples were composited for each ewe lamb and analyzed for N content using Kjeldahl procedure. Nitrogen intake was calculated by multiplying DM intake by the N content of each diet. Nitrogen lost in feces and urine was calculated by multiplying N content in the samples by fecal and urinary output, respectively. Then, retained N (g/day) was calculated by subtracting fecal and urinary output from the N intake. Nitrogen retention (%) was calculated by dividing the retained N by N intake.

Using the ewe lamb as the random variable and the treatment as the fixed effect, all data were analyzed using the MIXED procedure of SAS (version 8.1, 2000, SAS Institute Inc., Cary, NC). To further identify significant differences among means, which was considered at (*p* ≤ 0.05), least square means of the MIXED procedures of SAS were used.

### 2.2. Experiment 2

To ensure lambing consistency among the experimental animals, sixty ewes were estrually synchronized using intravaginal sponges. Based on the lambing dates, thirty Awassi ewes with similar initial body weight (43.2 ± 0.97 kg), age (3 to 4 years), and nursing single lambs were selected for the experiment and then randomly assigned into the three diets (10 ewes/diet). Ewes were housed individually in shaded pens (0.75 × 1.5 m) starting on day 2 postpartum. The shaded pens contained plastic feed and water buckets. Diets were balanced to fit the requirement of nursing ewes [10]. Ewes feed was offered *ad libitum* at 8:00 am, allowing 10% refusal with free access to water throughout the experiment. On a daily basis, nutrient intake was evaluated. During the study, lamb intake was solely dependent on ewe’s milk. After the end of the study lambs started to be fed concentrate and roughages, where the weaning process started. The study lasted for 8 weeks; the first week was used as an adaptation period for the diets and pens, and data were collected on the subsequent seven-week period of the study. Feed refusals were weighed, recorded, and sampled daily to accurately measure nutrient intakes. Animals body weights were measured at the commencement of the study and biweekly, thereafter, during the experiment. Milk yield was recorded at 8:00 h on a biweekly basis by hand milking. The milking process was performed by trained personnel, where lambs were separated from their dams 12 h before the hand milking. Milk yield was calculated over a 24-h period. A sample of 125 mL was collected from each ewe and analyzed directly for total solids using a forced oven set at 55 °C, fat using the Gerber method (Gerber Instruments, K. Schnider and Co. AG; 8307 Langhag, Effretikon, Switzerland), and Crude protein (N × 6.38) using the Kjeldahl procedure.

To determine the chemical composition of the diets and feed refusal, samples were dried in a forced air oven at 105 °C overnight to determine final DM [11]. Crude protein (N × 6.25) was measured using the Kjeldahl procedure. Neutral detergent fiber and acid detergent fiber were analyzed using an ANKOM^2000^ fiber analyzer (ANKOM technology Corp., Fairport, NY, USA). Ether extract was evaluated using Soxtec procedure (SXTEC SYSTEM HT 1043 Extraction Unit, TECATOR, Box 70, Hoganas, Sweden). Dry matter and other nutrient intakes were determined by subtracting refused feed from offered feed. Metabolizable energy was calculated using the tabular values of NRC [10].

Data were analyzed as a completely randomized design using the MIXED procedure of SAS (version 8.1, 2000, SAS Inst. Inc., Cary, NC, USA). Milk production and ewe and lamb BW were analyzed by analysis of variance for repeated measures in a model, including treatment, week, and the treatment × week interaction (i.e., week 2, 4, 6, 8). Because no treatment × week interaction was detected, only the main effects are discussed. For diets, milk composition, milk yield, nutrient intakes, ewe and lamb BW are discussed; the only fixed effect was treatment. A probability of *p* ≤ 0.05 was considered as a significant difference.

## 3. Results

The inclusion of SM decreased the cost of diet by 11 and 22% for the SM7.5 and SM15 diets, respectively, compared with the SM0 diet (Table 1). Chemical composition of the diets is presented in Table 1. The diets were formulated by partial or complete replacement of SBM with SM. The inclusion of SM did not change the nutrient content, except for the EE content, which was higher in the SM-containing diets compared with the control.

### 3.1. Experiment 1

Nutrient intakes and digestibility were similar among dietary treatments, except for EE intake and digestibility, which were greater (*p* < 0.001) in the SM-containing diets compared with the SM0 diet (Table 2). Nitrogen intake and N lost in the feces were not affected by treatment. However, N lost in the urine was greater (*p* = 0.003) in the SM7.5 than in the SM0 and SM15 diets. Retained N (g/d) and retention percentage were lower (*p* ≤ 0.05) in the SM7.5 than in the SM0 and SM15 diets (Table 2).

### 3.2. Experiment 2

The inclusion of SM did not affect nutrient content of the dietary treatments, except for EE content, which increased as the level of SM inclusion increased. Intakes of DM, CP, NDF, and ADF were similar among the dietary treatments (Table 3), however, EE intake was the highest (*p* < 0.0001) in SM15 followed by SM7.5 and SM0 diets.

Initial and final BW of ewes was similar among dietary treatments (Table 4). However, ewes fed the SM0 diet tended to lose less BW (*p* = 0.09) when compared with ewes fed the SM7.5 and SM15 diets. Lamb weaning weights, total gain, and pre-weaning average daily gain were greater (*p* ≤ 0.05) in SM-containing diets compared with SM0 diet.

Milk yield was greater (*p* = 0.05) in SM7.5 and SM15 diets than in the SM0 diet (Table 5). No significant differences were observed in milk total solids and fat content, however, protein content, daily total solids, and daily protein yield were greater (*p* ≤ 0.05) in the SM-containing diets than in the SM0 diet. Cost/kg of milk production was greater (*p* < 0.05) in SM0 vs. SM-containing diets. Additionally, milk production efficiency (DM intake: milk production) was improved (*p* < 0.05) in ewes fed the SM-containing diets compared with the controls.

## 4. Discussion

It is worth mentioning that there are 36 factories in Jordan that produce Tahini (Sesame paste), and the SM is the common by-product for the Tahini producing process. In such cases, our study can benefit the farmers, as SM is available on the market and is cheaper than SBM, in addition to reducing the cleaning and waste disposal costs for factories. This study showed that the use of SM at 7.5 and 15% of the dietary DM decreased the cost of diets by 11 and 22% for the SM7.5 and SM15 diets than the SM0 diet (Table 1). Similar data were reported by our research team when SBM was replaced by SM and sesame hulls [1,12].

No differences in nutrient composition of the three diets were observed except for EE, which was due to the greater level of EE in SM (11.9% EE) compared with SBM (1.2% EE). Consistent with previous studies [13,14], CP content of SM was comparable with that used in our study. However, other studies reported lower CP content [15,16]. The method of the oil extraction could be the reason for this discrepancy, assuring the necessity of analyzing the nutrient content of SM, regardless of source, before being incorporated into the ration.

In the first experiment, intake and digestibility of DM, CP, NDF, and ADF were not affected by the replacement of SBM with SM, while EE intake and digestibility were improved when ewes consumed SM. The greater EE intake and digestibility for ewes fed SM-diets in this study is due to the high level of EE in SM when compared to SBM. Obeidat et al. [1] found similar results in lambs fed 16% SM, but EE digestibility was not significantly different, whereas it tends to increase with SM amount into the diet. On the other hand, Ghorbani et al. [4] found that DM, CP, and EE intake were similar for lambs fed different levels of SM, while finding an increase of DM digestibility or no difference when SM increased in the diets. Consequently, present authors found similar results on DM and EE digestibility. Differences in intake and digestibility of nutrients in the aforementioned studies may be related to differences in ration composition and the nutrient content of the sesame meal (as affected by the method of oil extraction). In the present study, nitrogen intake, balance, and loss were not affected by the different treatments. These results are similar to results obtained by Obeidat et al. [1].

During the second experiment, ewe body weights were slightly reduced by consuming the SM-containing diets when compared to the SM0 diet. This result is inconsistent with the results reported by Bushara et al. [7], who found that body weight of adult does improved when consuming different levels of residual sesame capsules compared with does consuming the control diet during pregnancy and lactation. They attributed these results to adequate energy supplementation, which allows for the storage of energy, resulting in better body weight, reducing the negative effects of fat mobilization during lactation, and greater milk production and weight gain [17]. In the current study, the slight weight loss of dams consuming SM might be due to transforming energy obtained from feed to the milk production process. This is further supported by the fact that suckled lambs in the SM7.5 and SM15 groups had improved pre-weaning and weaning weights compared with those in the control group. Similar results were reported by other studies [14,18], whereby lambs of dams fed high protein and energy diets (such as SM containing diets) had improved body weights and linear measurements.

In the current study, using SM as total or partial replacement of SBM improved milk production and pre-weaning lamb growth and reduced the cost of milk production. Similar results were reported by Hejazi and Abo Omar [19], who found that milk yield increased more in goats consuming diets with 10% and 15% sesame oil cake than in goats fed diets 5% sesame oil cake. They also reported an increase in milk fat, milk protein, and total solids obtained from goats consuming diets with 15% sesame oil cake. In the current study, total milk solids and fat were not affected by the level of SM incorporation, while milk protein content and yield improved in ewes fed SM-containing diets.

The increase in milk yield and composition in the groups supplemented with SM may be attributed to the higher level of energy fed to those groups compared with the SM0 group, as shown in Table 1 (2.63, 2.69, or 2.75 Mcal/kg of dietary DM for the CON, SM7.5, or SM15, respectively). The dietary concentrate level and the composition of specific concentrates and grains impact the level of milk production and composition [20]. Caja and Bocquier [21] reviewed the effect of nutrition level on milk composition. The authors reported negative effects of energy balance on milk fat and positive effects on milk protein. They also concluded that high level of nutrition will decrease fat and slightly increase milk protein in most cases in dairy sheep. Increasing dietary protein concentration in well-fed ewes showed no effect on milk fat or milk protein contents; although many studies showed that milk yield and concentration of milk fat can be increased by increasing the protein content of the diet [22,23].

Sesame meal addition to the ration of lactating ewes resulted in a reduction of milk production cost (per Kg) when compared with SBM. This result is compatible with that reported by other researchers [1,7,12,19]. All previous studies revealed a positive economic benefit of adding sesame meal to their livestock diets by reporting a reduction in production cost and an increase in profitability. Generally speaking, the price of soybean meal is higher than that of sesame meal. Therefore, in the current study, partially or fully replacing soybean meal by sesame meal resulted in about 15% reduction in production cost. As a result, substitution of soybean meal with sesame meal reduced the cost of milk production, improved profitability, and enhanced the ewes’ feed efficiency.

## 5. Conclusions

Results of the current study demonstrate the effectiveness of using sesame meal as an alternative protein feedstuff to replace half (7.5% inclusion) or all (15% inclusion) the soybean meal (the most expensive conventional ingredient in ruminant diets) content in the diets of lactating Awassi ewes. This is primarily because nutrient content, intake, and digestibility of sesame meal are similar to those in soybean meal. Such replacement resulted in comparable animal productivity among groups receiving the different protein sources, while the cost of production was lower in animals receiving the SM-containing diets. Additionally, the current results are valuable in terms reducing waste generated by the sesame seed oil industry.

## Figures and Tables

**Table 1:** Ingredients and chemical composition of diets fed to Awassi ewes (Experiment 1 and 2).

	Diets *		
	SM0	SM7.5	SM15
Ingredients (% DM)			
Barley grain	58.0	58.0	58.0
Soybean meal, 44% CP (solvent)	15.0	7.5	0
Sesame meal	0	7.5	15.0
Wheat straw	26.0	26.0	26.0
Salt	0.5	0.5	0.5
Limestone	0.5	0.5	0.5
Mineral vitamin premix **	+	+	+
Feed cost / ton (US$)	334	297	261
Nutrients			
Dry matter, % **DM**	90.0	90.1	90.0
Crude protein, % **DM**	16.1	16.1	16.1
Neutral detergent fiber, % **DM**	31.1	30.9	30.7
Acid detergent fiber, % **DM**	17.6	17.5	17.4
Ether extract, % **DM**	2.6	4.3	6.0
Metabolizable energy, Mcal/kg	2.64	2.69	2.71

Note: * Dietary treatments are (1) no sesame meal (SM0), (2) 7.5% sesame meal (SM7.5), or (3) 15% sesame meal (SM15) of dietary dry matter (DM) in substitution of soybean meal; ** composition per kg contained (vitamin A, 600,000 IU; vitamin D3, 200,000 IU; vitamin E, 75 mg, vitamin K3, 200 mg; vitamin B1, 100 mg; vitamin B5, 500 mg; lysine 0.5%; DL-methionine, 0.15%; manganese oxide, 4000 mg; ferrous sulphate, 15,000 mg; zinc oxide, 7000; magnesium oxide, 4000 mg; potassium iodide, 80 mg; sodium selenite, 150 mg; copper sulphate, 100 mg; cobalt phosphate, 50 mg, dicalcium phosphate, 10,000 mg.

**Table 2:** Effect of feeding sesame meal (SM) on nutrient intake, digestibility, and N balance in Awassi ewe lambs (Experiment 1).

	Diets *			
Item	SM0 (*n* = 6)	SM7.5 (*n* = 6)	SM15 (*n* = 6)	SEM
**Intake, g/d**				
Dry matter	1368	1213	1256	54.5
Crude protein	218	193	202	8.8
Neutral detergent fiber	475	432	442	19.3
Acid detergent fiber	205	197	211	9.0
Ether extract	29 ^a^	51 ^b^	87 ^c^	3.1
**Digestibility %**				
Dry matter	78.1	73.4	75.2	1.58
Crude protein	79.5	76.0	79.2	1.34
Neutral detergent fiber	61.8	54.2	56.7	3.11
Acid detergent fiber	57.0	50.6	52.9	2.55
Ether extract	78.0 ^a^	86.9 ^b^	92.9 ^c^	1.91
**N Balance**				
N intake, g/d	38.4	33.4	35.5	2.21
N feces, g/d	8.3	8.6	7.6	0.68
N urine, g/d	7.0 ^a^	9.3 ^b^	5.7 ^a^	0.59
N retained, g/d	23.8 ^b^	16.2 ^a^	22.7 ^b^	1.83
N retention, %	61.9 ^b^	48.9 ^a^	63.3 ^b^	2.22

Note: * Dietary treatments are (1) no sesame meal (SM0), (2) 7.5% sesame meal (SM7.5), or (3) 15% sesame meal (SM15) of dietary dry matter (DM) in substitution of soybean meal; ^a,b,c^ within a row means without a common superscript difference (*p* ≤ 0.05).

**Table 3:** Effect of feeding sesame meal (SM) on nutrient intake of Awassi ewes fed lactation diets (Experiment 2).

	Diets *			
Item	SM0 (*n* = 10)	SM7.5 (*n* = 10)	SM15 (*n* = 10)	SEM
**Nutrient intake, g/d**				
Dry matter	2039	1984	1962	45.51
Crude protein	329	319	316	7.33
Neutral detergent fiber	634	613	602	14.08
Acid detergent fiber	359	348	342	8.00
Ether extract	54 ^a^	85 ^b^	116 ^c^	1.95

Note: * Dietary treatments are (1) no sesame meal (SM0), (2) 7.5% sesame meal (SM7.5), or (3) 15% sesame meal (SM15) of dietary dry matter (DM) in substitution of soybean meal fed for 8 weeks; ^a,b,c^ within a row means without a common superscript difference (*p* ≤ 0.05).

**Table 4:** Effect of feeding sesame meal (SM) on ewes’ body weight change and growth rate of their lambs (Experiment 2).

	Diets *			
Item	SM0 (*n* = 10)	SM7.5 (*n* = 10)	SM15 (*n* = 10)	SEM
**Ewes**				
Initial body weight (kg)	43.0	43.6	43.0	0.97
Final body weight (kg)	41.1	40.8	40.1	1.02
Body weight change, kg	−1.9	−2.9	−2.9	0.39
**Lambs**				
Initial body weight (kg)	5.0	4.8	4.7	0.21
Final body weight (kg)	18.3 ^a^	19.4 ^b^	20.0 ^b^	0.29
Total gain (kg)	13.3 ^a^	14.6 ^b^	15.3 ^b^	0.30
Average daily gain (g/d)	238 ^a^	261 ^b^	273 ^b^	5.3

Note: * Dietary treatments are (1) no sesame meal (SM0), (2) 7.5% sesame meal (SM7.5), or (3) 15% sesame meal (SM15) of dietary dry matter (DM) in substitution of soybean meal fed for 8 weeks; ^a,b^ within a row means without a common superscript difference (*p* ≤ 0.05).

**Table 5:** Effect of feeding sesame meal (SM) on milk yield, milk composition, and yield of milk component of Awassi ewes fed lactation diets (Experiment 2).

	Diets *			
Item	SM0 (*n* = 10)	SM7.5 (*n* = 10)	SM15 (*n* = 10)	SEM
Milk yield, g/d	1001 ^a^	1235 ^b^	1266 ^b^	80.8
Milk composition, %				
Total Solids	17.8	18.5	19.1	0.61
Protein	4.0 ^a^	4.5 ^b^	4.5 ^b^	0.19
Fat	6.8	6.7	7.2	0.31
Milk component yield, g/d				
Total Solids	177.4 ^a^	229.3	244.9	17.45
Protein	39.3 ^a^	55.3 ^b^	58.0 ^b^	3.71
Fat	67.9 ^a^	83.3 ^ab^	91.5 ^b^	7.27
Cost/kg milk production (US$)	0.72 ^a^	0.49 ^b^	0.43 ^b^	0.053
Feed efficiency **	2.15 ^b^	1.65 ^a^	1.64 ^a^	0.23

Note: * Dietary treatments are (1) no sesame meal (SM0), (2) 7.5% sesame meal (SM7.5), or (3) 15% sesame meal (SM15) of dietary dry matter (DM) in substitution of soybean meal fed for 8 weeks; ** feed efficiency (DM intake: milk production); ^a,b^ within a row means without a common superscript difference (*p* ≤ 0.05).
